# Mental health, risk perception, and coping strategies among healthcare workers in Egypt during the COVID-19 pandemic

**DOI:** 10.1371/journal.pone.0282264

**Published:** 2023-02-27

**Authors:** Mohamed E. G. Elsayed, Radwa Abdullah El-Abasiri, Roy Rillera Marzo, Khaled T. Dardeer, Manar Ahmed Kamal, Heba Abdelaziz, Soliman Belal Soliman, Mila Nu Nu Htay

**Affiliations:** 1 Department of Psychiatry and Psychotherapy III, University of Ulm, Ulm, Germany; 2 Department of Psychiatry, School of Medicine and Health Sciences, Carl von Ossietzky University Oldenburg, Oldenburg, Germany; 3 Nuffield Department of Population Health, Old Road Campus, University of Oxford Richard Doll Building, Oxford, United Kingdom; 4 Department of Community Medicine, International Medical School, Management and Science University, Selangor, Malaysia; 5 Global Public Health, Jeffrey Cheah School of Medicine and Health Sciences, Monash University Malaysia, Subang Jaya, Malaysia; 6 Institute of Applied Health Sciences, School of Medicine, Medical Sciences & Nutrition, University of Aberdeen, Aberdeen, United Kingdom; 7 Faculty of Medicine, Cairo University, Cairo, Egypt; 8 Faculty of Medicine, Benha University, Benha, Egypt; 9 Public Health Department, The National Hepatology and Tropical Medicine Research Institute, Cairo, Egypt; 10 Critical Care Medicine, Faculty of Medicine, Cairo University, Cairo, Egypt; 11 Department of Community Medicine, Faculty of Medicine, Manipal University College Malaysia, Melaka, Malaysia; King Khalid University, EGYPT

## Abstract

**Background:**

Coronavirus disease-19 emerged in December 2019. Healthcare workers were exposed to this highly infectious virus during the pandemic and suffered several social and psychological consequences, such as anxiety, psychological distress, and burnout.

**Objectives:**

To assess the psychological distress, anxiety, depression, coping strategies, risk perception, and attitude toward interprofessional teamwork among Egyptian healthcare workers during the COVID-19 pandemic.

**Methods:**

We conducted a cross-sectional online survey which consisted of five sections. The primary outcomes were anxiety (GAD-7), depression (PHQ-9), risk perception towards COVID-19, interprofessional teamwork attitude, and coping strategies during the Coronavirus disease-19 pandemic. The web-based questionnaire was distributed to Egyptian healthcare workers from the 20^th^ of April 2020 to the 20^th^ of May 2020. A snowball sampling method was used. Regression analysis was conducted to test the relationship between the socioeconomic characteristics and the previously mentioned outcomes.

**Results:**

A total of 403 participants responded to the online questionnaire. The majority were females (70.5%) and within the age group of 26–40 years (77.7%), with 2–5 years of work experience (43.2%). Most participants were pharmacists (33%) and physicians (22.1%). Eighty-two participants (21%) reported moderate to severe anxiety, and 79 participants reported (19.4%) moderate to severe depressive symptoms. In the univariate model, the marital status was associated with depression (OR 0.47, 95% CI 0.28–0.78), anxiety (OR 0.52, 95% CI 0.32–0.85), and an attitude toward interprofessional teamwork (β = -1.96 95% CI -2.72 to -1.2). Providing direct care to the patients was associated with lower anxiety symptoms (AOR 0.256, 95% CI 0.094–0.697). More severe anxiety and depressive symptoms were associated with difficulties in everyday life and the professional work environment (AOR 4.246 and 3.3, *P* = 0.003 and 0.01, respectively). Availability of mental health facilities at the workplace was associated with a lower risk perception towards COVID-19 (β = -0.79, 95% CI -1.24 to -0.34) and a more positive attitude towards teamwork (β = 2.77 95% CI 1.38–4.15).

**Conclusions:**

According to our results, the COVID-19 pandemic was associated with mild anxiety and depression among healthcare workers in Egypt, especially pharmacists and physicians. We recommend more research targeting the mental health of healthcare workers in Egypt. If proven cost-effective and needed, wide-scale mental health screening and public health campaigns can facilitate effective prevention and treatment strategies. In addition, the availability of mental health facilities at the workplace could alleviate some of the risk perception associated with health emergencies and improve interprofessional teamwork.

## Introduction

The severe acute respiratory syndrome coronavirus 2 (SARS-CoV-2) emerged in December 2019 and spread worldwide, causing the Corona Virus Disease 2019 (COVID-19) pandemic [1]. Coronaviruses induce respiratory tract infections in humans, such as Middle East respiratory syndrome (MERS) [2] and Severe Acute Respiratory Syndrome (SARS) [3]. Before the development of effective vaccines, healthcare workers’ high infection and mortality rates were an issue of great concern during the COVID-19 pandemic [4, 5]. Furthermore, many infected patients overwhelmed the healthcare system in many countries leading to severe stress affecting the healthcare workers physically and mentally [5–7]. Healthcare workers were reported to be more prone to COVID-19 infection, burnout, distress, and fear of transmitting the virus to their families and friends [5–9].

Previous literature showed that the COVID-19 pandemic worsened the general psychological symptoms [10] and several social, economic, and physiological effects [11]. COVID-19 also led to long-term mental health impacts [12]. Mental health worsened during the pandemic, especially among younger individuals with low income or a history of depression or anxiety [13]. Further risk factors of the mental health worsening were facing a financial impact, employment changes, smoking, increased alcohol intake over the preceding six months, having multiple comorbidities, or being in contact with COVID-19 cases [14]. Frequent exposure to social media news concerning COVID-19 was also considered a significant risk factor [15]. Additionally, healthcare workers from Middle- and low-income countries often suffered from significant psychological effects of the COVID-19 pandemic due to pre-existing risk factors, such as low compensation, fragmented infrastructure, and shortage of mandatory resources such as personal protective equipment (PPE) [16].

A study in China examined more than 1200 healthcare workers during the COVID-19 pandemic. It concluded that anxiety, depression, and distress were highly prevalent in the studied population with 45%, 50%, and 72%, respectively [1]. Another study conducted in India investigated the physicians’ attitudes and concluded that difficult work conditions and a shortage of PPE predicted a higher level of mental distress during the pandemic [9].

A review from Brooks et al. about the psychological consequences of the lockdown showed negative symptoms, such as anxiety, insomnia, stress, depression, confusion, post-traumatic stress symptoms, and anger [17]. Various studies were conducted, especially in China, to investigate the impact of the COVID-19 pandemic on the population’s mental health, the risk perception of being infected, and the coping strategies of healthcare workers [1]. Recent literature showed the psychological impacts of COVID-19 on healthcare workers’ mental health in western communities. A previous study observed that having a friend who died from COVID-19 or having higher COVID-19 worry scores were factors significantly associated with higher scores of distress [18].

Mitigation of the harmful effects of COVID-19 on mental health is considered an international public health priority [15]. However, there is limited evidence regarding the psychological distress during the pandemic amongst community healthcare workers in Middle Eastern countries such as Egypt. A study conducted among healthcare professionals in Egypt assessed the mental health consequences and reported anxiety (42.6%), depression (59.0%), and insomnia (51.9%) [19]. In this study, most respondents (77%) were physicians. Another study investigated mental well-being among frontline healthcare workers in Egypt and reported anxiety (76.4%) and depressive symptoms (77.2%) [20]. To deal with healthcare workers’ challenges, coping strategies, teamwork, and resilience were essential during the pandemic. The impact of stress on healthcare workers’ mental health was highly dependent on their cultural background. Investigations in different countries were needed; as the COVID-19 pandemic spread globally. The early detection of psychological distress among healthcare employees and the offering of mental health support for them was needed to ensure the functioning of healthcare systems. Therefore, this study aims to provide evidence for risk perception and coping strategies among Egyptian healthcare workers during the COVID-19 pandemic, assesses the prevalence of anxiety, depression, and risk perception among healthcare workers, as well as the attitude toward interprofessional teamwork in the workplace.

## Materials and methods

### Study design

This cross-sectional study was conducted by recruiting healthcare workers living and working in Egypt as a subset of a global study [21].

The participants’ informed consent was obtained before voluntary and confidential participation. Researchers collected the data using an online survey form distributed using different social media platforms (Twitter, Facebook, and invitation through email).

### Study participants and sample size

Data collection was conducted for one month, from the 20^th^ of April 2020 to the 20^th^ of May 2020. Participants were recruited from various healthcare professions, including physicians, pharmacists, nurses, medical assistants, laboratory technicians, and public health practitioners working in the governmental and private healthcare sectors. As only minimal literature on the topic was available at the start of this research project (in the early stage of the pandemic), the available study that investigated mental health outcomes among healthcare workers in China was utilized as a rough estimate in sample size calculation [1]. The sample size was estimated with an infinite population, a Z score of 1.96, a confidence level of 95%, an alpha level of 0.05, and a prevalence of relevant outcomes (anxiety and depression) of around 50% according to available literature at that time [1]; this gives a sample size of 385.

### Ethical consideration

Ethical approval was granted from the Research Ethics Committee of Asia Metropolitan University (AMU) in Johor, Malaysia, Project Ref No: AMU/MREC/FOM/NF/03/2020. The participants’ written consent was obtained before participation.

### Study instrument

The online survey included five sections as follows: 1) demographic characteristics: Participants were asked to enter their nationality, gender, age, religion, marital status (married/single/divorced/separated or widowed), their living conditions (living alone/with family/with friends), their current occupation, duration of working experience, and their current workplace. Participants were also asked if they worked in the intensive care Unit (ICU), provided healthcare directly to patients, or had contact with confirmed or suspected COVID-19 cases at their workplace. 2) the 7-item Generalized Anxiety Disorder (GAD-7) scale [22]. 3) the 9-item Patient Health Questionnaire (PHQ-9) [23]. 4) the risk perception questions adapted from Dai Y et al. [24]. 5) Questions regarding attitudes towards interprofessional teamwork adapted from Htay et al. 2021 [25]. 6) Multiple response items and an open question regarding coping strategies among healthcare workers [26].

### Anxiety assessment (GAD-7 score)

The Generalized Anxiety Disorder 7-item (GAD-7) questionnaire was utilized to assess the general anxiety symptoms among the participants. Anxiety severity was scored on a four-point Likert scale ("Not at all = 0", "Several days = 1", "More than half of the days = 2", and "Nearly every day = 3") with total scores ranging from 0 to 27. Cut points 5, 10, and 15 were suggested to represent mild, moderate, and severe anxiety levels on the scale [22]. The Internal consistency of this tool was satisfactory (Cronbach’s alpha 0.896) [27].

### Depressive symptoms assessment (PHQ-9 score)

The nine-item Patient Health Questionnaire (PQ-9) was used to assess the depression level among study participants. Each item of the questionnaire was scored on a four-point Likert scale ("Not at all = 0", "Several days = 1", "More than half of the days = 2", and "Nearly every day = 3") with the total score ranging from 0 to 27. Cut points 5, 10, and 15 were suggested to represent mild, moderate, and severe levels of depression on the scale [23]. Cronbach’s alpha of 0.896 was found for this tool, which is highly satisfactory [27].

### Concerns about COVID-19 (risk perception score)

The risk perception towards COVID-19 was assessed by six statements with three possible responses "Agree," "Neutral," and "Disagree". The six statements were; 1) I am worried about getting infected with COVID-19; 2) I am worried about my family members getting COVID-19 from me; 3) I am worried about my colleagues (in the team) getting infected with COVID-19; 4) I am worried about inadequate personal protective equipment for healthcare personnel (PPE); 5) I am worried about fake news which might be spreading out in the community; 6) I am worried about the prevention and control measures that we are practicing at the current moment. Responses were coded as "Agree = 2", "Neutral = 1", and "Disagree = 0". A total score of 12 was possible [28]. Cronbach’s alpha was 0.897 for this tool [27].

### Attitude towards interprofessional teamwork in the workplace (teamwork score)

Participants were asked to respond to five statements about their attitudes regarding interprofessional teamwork. The five statements were; 1) Interprofessional teamwork and collaboration reduce stress in managing the patients during the pandemic. 2) I am willing to discuss patient management with my team during the pandemic. 3) Disagreement (or) arguments often occur in my team, which remains unsolved; 4) Interprofessional teamwork improved the health outcomes of the patients during a pandemic; and 5) I am getting psychological support from team members at the workplace during a pandemic. The responses were coded as follows: (Strongly disagree = 1, disagree = 2, neutral = 3, agree = 4, strongly agree = 5). A reverse score was coded for the negative statement. A score of 25 was possible, with a higher score indicating a positive attitude toward interprofessional teamwork in the workplace (26). Cronbach’s alpha was below an acceptable level for this tool, 0.528.

### Coping strategies

To assess the coping strategies used during this period, participants were asked to select their methods to cope with the COVID-19 pandemic from available choices in the questionnaire. Participants were permitted to choose more than one single answer from 1) family support, 2) peer support, 3) religion, 4) exercise, 5) positive thinking, 6) mindfulness, 7) meditation, and 8) others (open answer). Comparisons were made between healthcare workers who provided direct care to patients and those who did not.

### Statistical analysis

IBM Statistical Package for the Social Sciences (SPSS) version 25.0 was used for the data analysis. Mean ± standard deviation (SD) or median and range are presented for continuous variables. Categorical variables are presented as numbers and percentages (%). Data normality was checked visually by inspecting histograms, Q-Q plots, and the Shapiro test. Four primary outcomes were assessed in this study: depression (PHQ-9 Score), anxiety (GAD-7 Score), concern towards covid (Risk perception score), and attitude towards interprofessional teamwork (Teamwork score). Depression and anxiety scores were categorized according to their respective guidelines. Risk perception and attitude towards interprofessional teams were used as continuous variables (no reasonable cut-off values were found in the literature for them). Logistic regression was conducted to determine the predictors for severe anxiety or depressive symptoms; a cut-off of 10 points was used to classify participants into low or mild versus severe symptomatology for the GAD-7 and PHQ-9 scores, respectively.

A general linear model (GLM) was conducted to test the associations between population characteristics, continuous risk perception variables, and attitudes toward teamwork. The univariate analysis examined the association of the characteristics of the population with depression, anxiety, risk perception, and attitude toward teamwork.

Multivariate analysis was conducted after adjusting for all other factors. Risk perception and teamwork score were used as continuous variables in the regression models, while all other factors were used as categorical variables. The results were presented as β coefficients with 95% confidence intervals (CI) for the GLM, odds ratios (OR), and adjusted odds ratios (AOR) with 95% CI for the logistic regression. Associations between the assessment tools were assessed using partial correlation after controlling for potential confounders. The internal consistency of the assessment tool was tested using Cronbach’s alpha [27]. Differences in coping strategies among participants were assessed using the Chi^2^ test. A *P*-value less than 0.05 was considered significant for all statistical tests.

## Results

### Characteristics of the study population

A total of 403 participants were included in this study. The majority were females (n = 284, 70.5%), in the age group 18–29 (n = 285, 70.7%), with 2–5 years of work experience (n = 174, 43.2%). The most common occupation among the study participants was being a pharmacist (n = 133, 33%), followed by being a university tutor (n = 96, 23.8%) and being a physician (n = 89, 22.1%). As for anxiety and depressive symptoms, most participants reported no to low symptoms as assessed by GAD-7 (n = 209, 51.9%) and PHQ-9 (n = 188, 46.7%) scores. Very few (13.4%) reported having difficulties in everyday life due to their anxiety and depressive symptoms **(Table 1)**.

**Table 1:** Sample characteristics.

Characteristics (n = 403)	No.	%
**Age (Mean ± SD)**	28.91 **±** 5.52	
**Age Groups**		
18–29 years	285	70.7
30–45 years	111	27.5
45–65 years	7	1.7
**Gender**		
Male	119	29.5
Female	284	70.5
**Religion**		
Islam	355	88.1
Christianity	48	11.9
**Marital status**		
Married	203	50.4
Single	200	49.6
**Work experience**		
< 2 years	103	25.6
2–5 years	174	43.2
6–10 years	78	19.4
> 10 years	48	11.9
**Living arrangement**		
Alone	31	7.7
With friends	21	5.2
With family	351	87.1
**Occupation**		
Pharmacist	133	33.0
Physician	89	22.1
Nurse	9	2.2
University Tutor	96	23.8
Medical Student	4	1.0
Medical Representative	29	7.2
Research scientist	6	1.5
Others*	36	8.9
**Current workplace (Multiple responses allowed)**		
Hospital	123	23.1
University	46	8.6
Home	131	24.6
Freelance	95	17.9
Pharmacy	47	8.8
Laboratory	10	1.9
Research Institute	7	1.3
Pharmaceutical Company	24	4.5
Clinic	29	5.5
Others**	20	3.8
**Do you work in the ICU?**		
No	329	81.6
Yes	74	18.4
**Do you provide direct care to patients?**		
No	223	55.3
Yes	180	44.7
**Direct contact with COVID-19 cases**		
No	292	72.5
Yes	110	27.3
**The mental health support team is available at my workplace**		
No	356	88.3
Yes	47	11.7
**I am getting support from the mental health support team at my workplace.**		
No	369	91.6
Yes	34	8.4
**Anxiety symptoms (GAD-7 Score)**		
No (0–4)	209	51.9
Mild (5–10)	107	26.6
Moderate (11–15)	55	13.6
Severe (> 15)	32	7.9
**Depressive symptoms (PHQ-9 Score)**		
No (0–4)	188	46.7
Mild (5–10)	136	33.7
Moderate (11–15)	50	12.4
Severe (> 15)	29	7.2
**If you checked off any problems, how difficult have these made it for you to do your work, take care of things at home, or get along with others?**		
Not difficult at all	187	46.4
Somewhat difficult	162	40.2
Very difficult	42	10.4
Extremely difficult	12	3.0
**Coping strategies reported by the participants**		
Positive thinking	266	66.8
Family support	248	62.3
Adequate Sleep	226	56.8
Religion	199	50.0
Pray	184	46.2
Watching TV	172	43.2
Learning new things	148	37.2
Peer support	61	15.3
Exercise	58	14.6
Work	52	13.1
Meditation	43	10.8
Video games	2	0.5

*Other occupations included lab technicians, HR employees, administrative jobs, sales and accounting jobs, and public health officers.

**Other workplaces included public health, sales management, insurance, and central administration of pharmaceutical affairs.

### Predictors of anxiety symptoms

Univariate analysis showed that married participants, people with less experience in the field, and university tutors significantly reported lower anxiety symptoms. Multivariable analysis showed that pharmacists reported lower anxiety levels than physicians. Study participants who provided direct care to patients reported lower anxiety levels than those who did not. On the other hand, higher depressive symptoms and positive attitudes toward interprofessional teamwork were significant predictors for higher anxiety among the study participants. No significant differences regarding age groups and gender were found in either the univariate or the multivariate models **(Table 2)**.

**Table 2:** Binary logistic regression explaining factors associated with higher anxiety among study participants.

Characteristics	Low anxiety		Moderate to severe anxiety		Unadjusted analysis				Adjusted analysis*			
	N	%	n	%	p	ORs	95	CI	P	ORs	95	CI
**Gender**												
Male	91	76.5	28	23.5	Ref				Ref			
Female	225	79.2	59	20.8	0.54	0.85	0.51	1.42	0.065	0.487	0.227	1.047
**Age Groups**												
18–29	221	77.5	64	22.5					Ref			
30–45	90	81.1	21	18.9	0.442	0.806	0.465	1.397	0.349	0.544	0.152	1.944
46–65	5	71.4	2	28.6	0.703	1.381	0.262	7.288	0.964	1.066	0.067	16.895
**Religion**												
Islam	274	77.2	81	22.8	Ref				Ref			
Christianity	42	87.5	6	12.5	0.109	0.48	0.2	1.18	0.542	0.683	0.200	2.328
**Marital status**												
Single	146	73	54	27	Ref				Ref			
Married	170	83.7	33	16.3	**0.009**	**0.52**	**0.32**	**0.85**	0.701	1.171	0.523	2.623
**Time on the job**												
< 2 years	71	68.9	32	31.1	Ref				Ref			
2–5 years	145	83.3	29	16.7	**0.006**	**0.44**	**0.25**	**0.79**	0.740	0.856	0.341	2.145
6–10 years	60	76.9	18	23.1	0.235	0.67	0.34	1.3	0.846	1.144	0.295	4.441
> 10 years	40	83.3	8	16.7	0.066	0.44	0.19	1.06	0.966	1.042	0.160	6.769
**Housing status**												
Alone	24	77.4	7	22.6	Ref				Ref			
With friends	20	95.2	1	4.8	0.112	0.17	0.02	1.51	0.446	0.364	0.027	4.901
With family	272	77.5	79	22.5	0.993	1	0.41	2.4	0.927	1.061	0.296	3.805
**Current occupation**												
Doctor	64	71.9	25	28.1	Ref				Ref			
Pharmacist	104	78.2	29	21.8	0.286	0.71	0.38	1.33	**0.037**	**0.342**	**0.124**	**0.939**
Nurse	7	77.8	2	22.2	0.708	0.73	0.14	3.76	0.980	1.033	0.075	14.210
Tutor	92	95.8	4	4.2	**<0.001**	**0.11**	**0.04**	**0.34**	0.081	0.228	0.044	1.197
Medical Rep	21	72.4	8	27.6	0.958	0.98	0.38	2.49	0.770	0.797	0.174	3.654
Others**	28	59.6	19	40.4	0.146	1.74	0.83	3.65	0.872	1.109	0.313	3.926
**Are you working in the ICU?**												
No	261	79.3	68	20.7	Ref				Ref			
Yes	55	74.3	19	25.7	0.345	1.33	0.74	2.38	0.802	0.876	0.310	2.473
**Do you provide direct care to the patients?**												
No	175	78.5	48	21.5	Ref				Ref			
Yes	141	78.3	39	21.7	0.973	1.01	0.63	1.63	**0.008**	**0.256**	**0.094**	**0.697**
**Are you in direct contact with COVID-19 cases?**												
No	233	79.8	59	20.2	Ref				Ref			
Yes	82	74.5	28	25.5	0.256	1.35	0.81	2.26	0.127	2.228	0.797	6.227
**The mental health support team is available at my workplace**												
No	283	79.5	73	20.5	Ref				Ref			
Yes	33	70.2	14	29.8	0.149	1.64	0.84	3.23	0.714	1.303	0.317	5.351
**I am getting support from the mental health support team at my workplace.**												
No	291	78.9	78	21.1	Ref				Ref			
Yes	25	73.5	9	26.5	0.471	1.34	0.6	2.99	0.915	1.090	0.223	5.318
**If you checked off any problems, how difficult have these made it for you to do your Work, care for things at home, or get along with others?**												
Not at all to somewhat difficult	291	83.4	58	16.6					Ref			
Highly or extremely difficult	25	46.3	29	53.7	**<0.001**	**5.820**	**3.179**	**10.654**	**0.003**	**4.246**	**1.628**	**11.074**
**Depressive symptoms (PHQ-9 Score)**												
Low (<10)	296	91.4	28	8.6	Ref				Ref			
High (>10)	20	25.3	59	74.7	**<0.001**	**31.19**	**16.47**	**59.04**	**<0.001**	**25.270**	**11.144**	**57.306**
**Risk perception (Score 0–12)** ***	-				0.096	1.17	0.97	1.41	0.060	1.257	0.990	1.594
**Attitude towards interprofessional teamwork (Score 5–25)** ***	-				**<0.001**	**1.200**	**1.129**	**1.276**	**0.004**	**1.159**	**1.050**	**1.280**

Anxiety symptoms: participants with GAD-7 score more than 10.

OR: Odds ratio, CI: Confidence interval

*Adjusted for age, gender, religion, marital status, work duration, living status, current occupation, working in the ICU, direct care to patients, direct contact with COVID patients, Available mental health support, using mental health support, Risk perception, teamwork score, and depressive symptoms (PHQ-9 score).

**Other occupations included lab technicians, HR employees, administrative jobs, sales and accounting jobs, and public health officers.

*** Score is a continuous variable; no categorization was done, and no groups were present.

### Predictors of depressive symptoms

Univariate analysis showed that married participants and university tutors reported lower depressive symptoms, such as anxiety. Multivariable logistic regression showed that participants with 2–5 years of experience were less likely to report more severe depressive symptoms than those with less than two years of work experience. In contrast, daily life problems due to concerns about COVID-19 and higher anxiety levels were significant predictors of depressive symptoms among the participants. Age and gender were not significant predictors for depression **(Table 3)**.

**Table 3:** Binary logistic regression explaining factors associated with higher depressive symptoms among study participants.

Characteristics	Low depressive symptoms		Moderate to Severe depressive symptoms		Unadjusted analysis				Adjusted analysis*			
	n	%	n	%	p	ORs	95	CI	p	ORs	95	CI
**Gender**												
Male	99	83.2	20	16.8	Ref				Ref			
Female	225	79.2	59	20.8	0.361	1.3	0.74	2.27	0.118	1.951	0.844	4.507
**Age Groups**												
18–29	226	79.3	59	20.7					Ref			
30–45	92	82.9	19	17.1	0.421	0.791	0.447	1.400	0.439	1.735	0.430	6.996
46–65	6	85.7	1	14.3	0.681	0.638	0.075	5.406	0.739	1.802	0.057	57.419
**Religion**												
Islam	281	79.2	74	20.8	Ref				Ref			
Christianity	43	89.6	5	10.4	0.095	0.44	0.17	1.15	0.262	0.464	0.121	1.775
**Marital status**												
Single	149	74.5	51	25.5	Ref				Ref			
Married	175	86.2	28	13.8	**0.003**	**0.47**	**0.28**	**0.78**	0.978	0.988	0.435	2.245
**Time spent on the job**												
< 2 years	69	67	34	33	Ref				Ref			
2–5 years	152	87.4	22	12.6	**<0.001**	**0.29**	**0.16**	**0.54**	**0.009**	**0.297**	**0.119**	**0.739**
6–10 years	60	76.9	18	23.1	0.145	0.61	0.31	1.19	0.320	0.488	0.119	2.006
> 10 years	43	89.6	5	10.4	**0.005**	**0.24**	**0.09**	**0.65**	0.097	0.175	0.022	1.368
**Housing status**												
Alone	26	83.9	5	16.1	Ref				Ref			
With friends	20	95.2	1	4.8	0.235	0.26	0.03	2.41	0.590	0.479	0.033	6.958
With family	278	79.2	73	20.8	0.538	1.37	0.51	3.68	0.991	0.992	0.235	4.179
**Current occupation**												
Physician	69	77.5	20	22.5	Ref				Ref			
Pharmacist	99	74.4	34	25.6	0.599	1.18	0.63	2.23	0.155	2.209	0.740	6.593
Nurse	7	77.8	2	22.2	0.986	0.99	0.19	5.12	0.337	3.487	0.272	44.626
Tutor	95	99	1	1	**0.001**	**0.04**	**0**	**0.28**	0.095	0.121	0.010	1.447
Medical Rep	22	75.9	7	24.1	0.853	1.1	0.41	2.94	0.209	2.620	0.582	11.787
Others**	32	68.1	15	31.9	0.233	1.62	0.73	3.56	0.451	1.707	0.425	6.854
**Are you working in the ICU?**												
No	270	82.1	59	17.9	Ref				Ref			
Yes	54	73	20	27	0.077	1.69	0.94	3.04	0.197	2.006	0.697	5.770
**Do you provide direct care to the patients?**												
No	185	83	38	17	Ref				Ref			
Yes	139	77.2	41	22.8	0.15	1.44	0.88	2.35	0.622	1.268	0.494	3.254
**Are you in direct contact with COVID-19 cases?**												
No	236	80.8	56	19.2	Ref				Ref			
Yes	87	79.1	23	20.9	0.697	1.11	0.65	1.92	0.220	0.505	0.169	1.506
**The mental health support team is available at my workplace**												
No	291	81.7	65	18.3	Ref				Ref			
Yes	33	70.2	14	29.8	0.065	1.9	0.96	3.75	0.812	1.172	0.318	4.315
**I am getting support from the mental health support team at my workplace.**												
No	299	81	70	19	Ref				Ref			
Yes	25	73.5	9	26.5	0.295	1.54	0.69	3.44	0.939	0.944	0.218	4.091
**If you checked off any problems, how difficult have these made it for you to do your work, take care of things at home, or get along with others?**												
Not at all to somewhat difficult	297	85.1	52	14.9					Ref			
Highly or extremely difficult	27	50.0	27	50.0	**<0.001**	**5.712**	**3.105**	**10.505**	**0.010**	**3.330**	**1.328**	**8.347**
**Anxiety symptoms (GAD—7 Score)**												
Low (<10)	296	93.7	20	6.3	Ref				Ref			
High (>10)	28	32.2	59	67.8	**<0.001**	**31.19**	**16.47**	**59.04**	**<0.001**	**28.160**	**12.182**	**65.100**
**Risk perception (Score 0–12)** ***	-				0.994	1	0.85	1.18	0.229	0.866	0.685	1.095
**Attitude towards interprofessional teamwork (Score 5–25)** ***	-				**<0.001**	**1.186**	**1.115**	**1.262**	0.239	1.065	0.959	1.182

Depressive symptoms: participants with PHQ-9 score more than 10.

OR: Odds ratio, CI: Confidence interval

*Adjusted for age, gender, religion, marital status, work duration, living status, current occupation, working in the ICU, direct care to patients, direct contact with COVID patients, Available mental health support, using mental health support, risk perception, teamwork score, and anxiety symptoms (GAD-7 Score).

**Other occupations included lab technicians, HR employees, administrative jobs, sales and accounting jobs, and public health officers.

***Score was treated as a continuous variable; no categorization was done, and no groups were present.

### Predictors of risk perception toward COVID-19

Univariate GLM analysis suggested some influence of gender and availability of mental health support at the workplace on participants’ risk perception towards COVID-19. However, following adjustment of other factors, anxiety was the only significant factor affecting the risk perception; participants with moderate or high anxiety had a mean score of 0.54 points higher on the risk perception score than those with low anxiety (β = 0.54, 95% CI 0.07–1.01, *P =* 0.023). Interestingly, providing direct care to patients and working in the ICU did not significantly affect the risk perception of the study participants **(Table 4)**.

**Table 4:** General linear model: Predictors of risk perception towards COVID-19.

Characteristics	Unadjusted analysis				Adjusted analysis*			
	B	95%	CI	P	B	95%	CI	P
**Gender**								
Male				Ref				Ref
Female	**0.34**	**0.02**	**0.66**	**0.038**	0.26	-0.06	0.59	0.113
**Age Groups**								
18–30				Ref				Ref
30–45	-0.29	-0.61	0.04	0.089	-0.09	-0.62	0.45	0.752
46–65	-0.31	-1.43	0.82	0.594	0.01	-1.25	1.27	0.99
**Religion**								
Muslim				Ref				Ref
Christian	-0.08	-0.53	0.37	0.731	0.02	-0.42	0.46	0.937
**Marital status**								
Single				Ref				Ref
Married	0.07	-0.22	0.37	0.619	0.05	-0.29	0.4	0.766
**Housing status**								
Alone				Ref				Ref
With friends	-0.5	-1.33	0.33	0.238	-0.57	-1.41	0.27	0.181
With family	0.09	-0.46	0.64	0.737	-0.04	-0.6	0.52	0.89
**Current occupation**								
Doctor				Ref				Ref
Pharmacist	-0.11	-0.51	0.29	0.6	-0.01	-0.46	0.44	0.97
Nurse	-0.44	-1.46	0.58	0.399	-0.33	-1.34	0.68	0.522
Tutor	0.11	-0.32	0.54	0.608	0	-0.61	0.62	0.994
Medical Rep	0.38	-0.24	1	0.232	0.39	-0.3	1.09	0.268
Others**	-0.44	-0.97	0.08	0.098	-0.56	-1.18	0.07	0.08
**Working duration**								
<2y				Ref				Ref
2-5y	0.11	-0.26	0.47	0.556	0.04	-0.35	0.43	0.84
6-10y	-0.24	-0.68	0.2	0.293	-0.27	-0.85	0.3	0.351
>10y	-0.31	-0.83	0.2	0.228	-0.35	-1.12	0.43	0.378
**Working in the ICU**								
No				Ref				Ref
Yes	-0.11	-0.49	0.27	0.575	-0.34	-0.81	0.13	0.159
**Providing direct care**								
No				Ref				Ref
Yes	-0.15	-0.45	0.14	0.305	-0.09	-0.52	0.34	0.676
**Contact with Covid case**								
No				Ref				Ref
Yes	0.09	-0.24	0.42	0.6	0.36	-0.09	0.82	0.119
**The mental health support team is available at my workplace**								
No				Ref				Ref
Yes	**-0.79**	**-1.24**	**-0.34**	**0.001**	-0.43	-1.05	0.19	0.175
**I am getting support from the mental health support team at my workplace.**								
No				Ref				Ref
Yes	**-0.97**	**-1.49**	**-0.45**	**0**	-0.7	-1.4	0.01	0.052
**If you checked off any problems, how difficult have these made it for you to do your**								
**work, take care of things at home, or get along with others?**								
Not at all to somewhat difficult				Ref				Ref
Highly or extremely difficult	0.03	-0.4	0.47	0.877	-0.14	-0.59	0.31	0.536
**Anxiety symptoms (GAD—7 Score)**								
Low (<10)				Ref				Ref
High (>10)	0.31	-0.05	0.66	0.092	**0.54**	**0.07**	**1.01**	**0.023**
**Depressive symptoms (PHQ-9 Score)**								
Low (<10)				Ref				Ref
High (>10)	-0.001	-0.371	0.368	0.994	-0.28	-0.75	0.2	0.259
**Attitude towards interprofessional teamwork (Score 5–25)** ***	-0.007	-0.041	0.027	0.7	0.02	-0.03	0.06	0.489

β: Beta coefficient, CI: Confidence interval, Ref: reference category

*Adjusted for age, gender, religion, marital status, work duration, living status, current occupation, working in the ICU, direct care to patients, direct contact with COVID patients, Available mental health support, using mental health support, teamwork score, anxiety symptoms (GAD-7 score), and depressive symptoms (PHQ-9 score).

**Other occupations included lab technicians, HR employees, administrative jobs, sales and accounting jobs, and public health officers.

***Score was treated as a continuous variable; no categorization was done, and no groups were present.

### Predictors of attitude towards interprofessional teamwork

**Fig 1:**
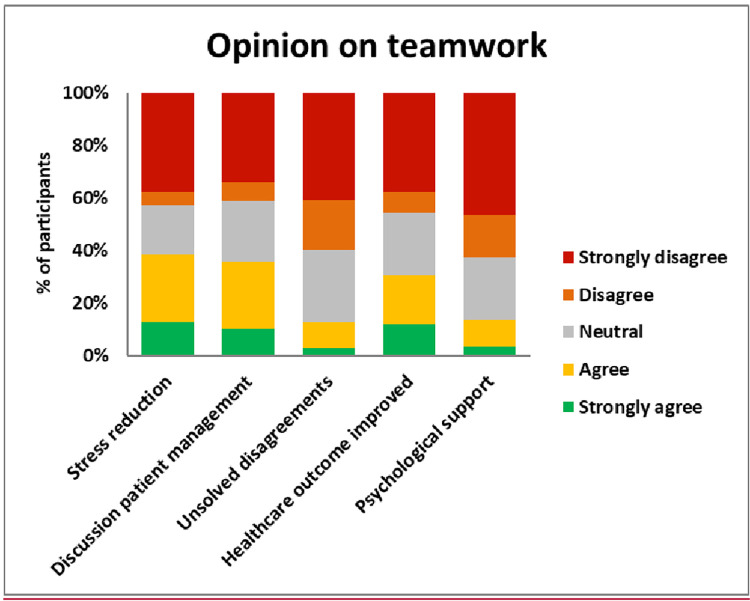
Opinions on interprofessional teamwork during the pandemic among healthcare workers.

The univariate model showed the effect of gender on interprofessional teamwork, with females having fewer positive attitudes. Multivariate GLM analysis revealed that being married, living with friends, working as a tutor or medical representative, and working in the ICU significantly influenced a negative attitude toward interprofessional teamwork. In contrast, longer duration of work, providing direct care to patients, availability of mental health support at the workplace, and, surprisingly, higher anxiety symptoms were significantly associated with a more positive attitude towards interprofessional teamwork. No significant association was found for gender in both models **(Table 5 & Fig 1)**.

**Table 5:** General linear model explaining factors associated with attitude towards interprofessional teamwork among study participants.

Characteristics	Unadjusted analysis				Adjusted analysis*			
	B	95%	CI	P value	B	95%	CI	P value
**Gender**								
Male				Ref				Ref
Female	-0.79	-1.71	0.14	0.096	-0.25	-0.99	0.49	0.513
**Age Groups**								
18–30				Ref				Ref
30–45	**0.97**	**0.03**	**1.92**	**0.044**	-0.42	-1.63	0.79	0.497
46–65	2.53	-0.7	5.76	0.125	-0.15	-3.01	2.71	0.917
**Religion**								
Muslim								
Christian	-1.17	-2.47	0.13	0.079	-0.44	-1.44	0.56	0.386
**Marital status**								
Single								
Married	**-3.25**	**-4.04**	**-2.47**	**<0.001**	**-1.96**	**-2.72**	**-1.2**	**<0.001**
**Living status**								
Alone				Ref				Ref
With friends	-2.13	-4.52	0.26	0.081	**-2.1**	**-3.99**	**-0.21**	**0.029**
With family	0	-1.58	1.59	0.995	0.81	-0.45	2.07	0.206
**Current occupation**								
Doctor				Ref				Ref
Pharmacist	0.18	-0.81	1.18	0.721	0.36	-0.66	1.38	0.486
Nurse	**-3.29**	**-5.84**	**-0.75**	**0.011**	-1.91	-4.19	0.36	0.1
Tutor	**-5.08**	**-6.15**	**-4.01**	**<0.001**	**-2.65**	**-4.02**	**-1.28**	**<0.001**
Medical Rep	**-2.67**	**-4.23**	**-1.12**	**0.001**	**-1.89**	**-3.46**	**-0.32**	**0.018**
Others**	0.43	-0.88	1.74	0.519	0.83	-0.59	2.24	0.251
**Working duration**								
<2y				Ref				Ref
2-5y	**-2.8**	**-3.8**	**-1.79**	**<0.001**	-0.44	-1.31	0.44	0.327
6-10y	-0.01	-1.23	1.2	0.981	**1.34**	**0.04**	**2.63**	**0.043**
>10y	-0.49	-1.9	0.93	0.501	1.64	-0.11	3.38	0.066
**Working in the ICU**								
No				Ref				Ref
Yes	0.17	-0.92	1.27	0.755	**-1.69**	**-2.74**	**-0.63**	**0.002**
**Providing direct care**								
No				Ref				Ref
Yes	**2.76**	**1.96**	**3.57**	**<0.001**	**1.28**	**0.31**	**2.25**	**0.01**
**Contact with Covid case**								
No				Ref				Ref
Yes	**1.41**	**0.46**	**2.35**	**0.003**	0.24	-0.79	1.28	0.644
**The mental health support team is available at my workplace**								
No				Ref				Ref
Yes	**3.5**	**2.23**	**4.78**	**<0.001**	**2.77**	**1.38**	**4.15**	**<0.001**
**I am getting support from the mental health support team at my workplace.**								
No				Ref				Ref
Yes	**1.91**	**0.4**	**3.43**	**0.013**	-1.56	-3.16	0.03	0.055
**If you checked off any problems, how difficult have these made it for you to do your**								
**work, take care of things at home, or get along with others?**								
Not at all to somewhat difficult				Ref				Ref
Highly or extremely difficult	**1.45**	**0.22**	**2.69**	**0.021**	0.44	-0.57	1.46	0.391
**Anxiety symptoms (GAD—7 Score)**								
Low (<10)				Ref				Ref
High (>10)	**3.26**	**2.28**	**4.24**	**<0.001**	**1.58**	**0.53**	**2.63**	**0.003**
**Depressive symptoms (PHQ-9 Score)**								
Low (<10)				Ref				Ref
High (>10)	**3.08**	**2.06**	**4.11**	**<0.001**	0.4	-0.68	1.49	0.466
**Risk perception (Score 0–12)** ***	-0.06	-0.34	0.23	0.701	0.08	-0.14	0.3	0.489

β: Beta coefficient, CI: Confidence interval

*Adjusted for age, gender, religion, marital status, work duration, living status, current occupation, working in the ICU, direct care to patients, direct contact with COVID patients, Available mental health support, using mental health support, risk perception score, anxiety symptoms (GAD-7 score), and depressive symptoms (PHQ-9 score).

**Other occupations included lab technicians, HR employees, administrative jobs, sales and accounting jobs, and public health officers.

***Score was treated as a continuous variable; no categorization was done, and no groups were present.

### Correlation within the assessment tools

After controlling for all other factors, anxiety was strongly correlated to depressive symptoms (r = 0.78, *P <* 0.001). The most surprising finding is that a more positive attitude toward interprofessional teamwork showed a negligible correlation with higher depressive symptoms (r = 0.262, *P <* 0.001) and a low correlation with anxiety symptoms (r = 0.38, *P <* 0.001). Risk perception was weakly correlated with anxiety symptoms (r = 0.11, *P* = 0.03) and not depressive symptoms. No significant correlation was found between risk perception toward COVID-19 and attitude toward teamwork (*P =* 0.41) **(Table 6)**.

**Table 6:** Partial correlations showing the associations between the assessment tools used in this study.

Variable*	Anxiety	Depressive symptoms	Risk perception	Positive attitude toward teamwork
Anxiety	1	**0.78** ***	**0.11** **	**0.38** ***
Depressive symptoms		1	0.04	**0.262** ***
Risk perception			1	0.04
Teamwork score				1

*Controlling for gender, age groups, religion, marital status, housing status, current occupation, time spent on the job, working in the ICU, providing direct care to patients, direct contact with COVID-19 cases, availability of mental health support in the workplace, using mental health support resources in the workplace

**Significant at p < 0.05 level

***Significant at p <0.001 level

### Coping strategies among study participants

**Fig 2:**
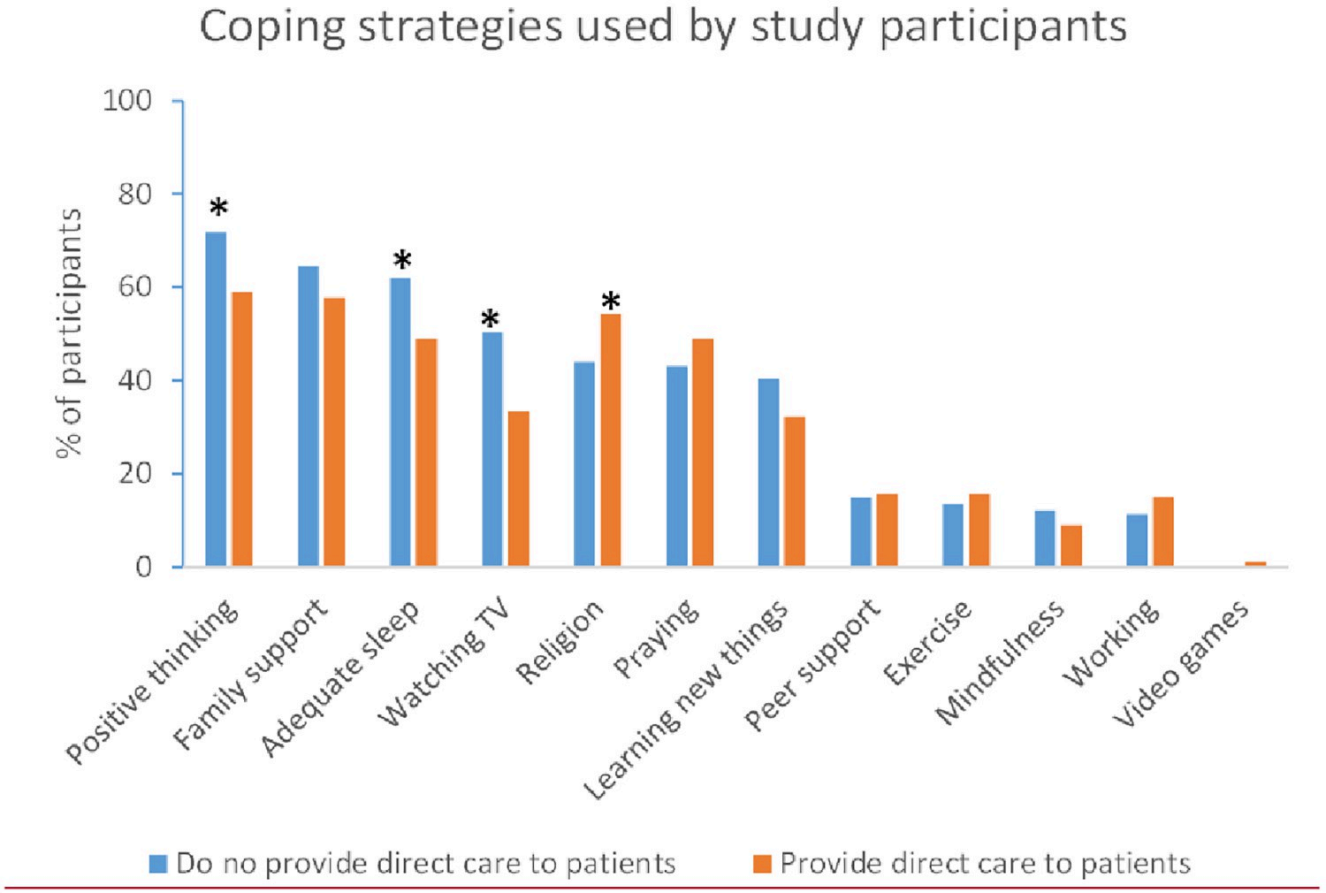
Coping strategies among participants who provide direct care to patients vs. those who do not.

**Fig 3:**
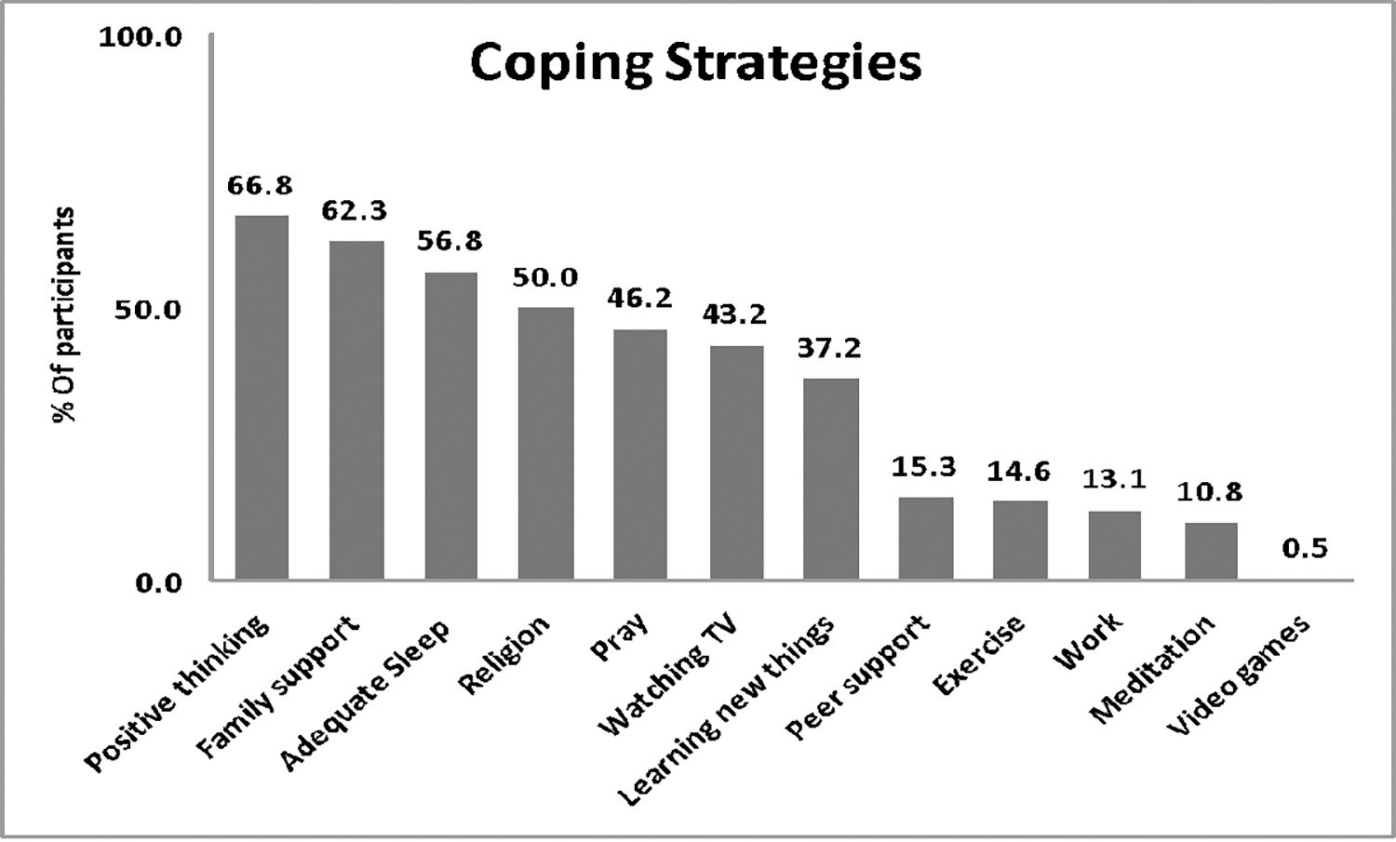
Coping strategies reported by the study participants during the COVID-19 pandemic.

Participants were asked to select all the methods they used to cope with the COVID-19 pandemic. Positive thinking was the most common choice (n = 266, 66.8%), followed by family support (n = 248, 62.3%) and adequate sleep (n = 226, 56.8%). Meditation (n = 43, 10.8%) and video games (n = 2, 0.5%) were the least choices. Most notably, coping strategies like adequate sleep (n = 138%), positive thinking (n = 160%), and watching TV (n = 112%) were reported more frequently by those who do not deal directly with patients **(Figs 2 & 3)**.

## Discussion

In December 2019, COVID-19 emerged and spread globally, causing a pandemic. The previous literature found that healthcare workers reported prevalent anxiety, depression, and distress. Our primary outcomes were designed to provide evidence for risk perception and coping strategies, estimate the prevalence of anxiety, depression, and risk perception among healthcare workers in Egypt, and identify the associated factors with mental health issues. Our main findings referred to the impact of the COVID-19 pandemic on mental health among healthcare workers in Egypt, especially pharmacists and physicians, in the form of mild levels of anxiety and depression. So, results were interpreted, discussed, and compared within the context of seven previous and similar studies through the country, total responses, age of the participants, gender, mental health assessment, used scale for mental health assessment, target population, and the main results. We summarized some of those points compared to the previous studies that estimated the prevalence of mental health issues among healthcare workers as Elsaie et al. (Egypt) [29], Elkholy et al. (Egypt) [20], Arafa et al. [30] (Egypt and Saudia Arabia), Alamri et al. (Saudi Arabia) [31], and Ghaleb et al. (Eastern Mediterranean Region) [18] **(S1 Table)**.

### Interpretation of sociodemographic characteristics

Regarding the sociodemographic characteristics of the participants, most participants in our study were from the age group of 26 to 40 years (70.7%), in agreement with almost all of the previous literature as Elsaie et al. (Egypt) (65.5%) [29], Elkholy et al. (Egypt) (44.4%) [20], Arafa et al. (Egypt and Saudi Arabia) (47.2%) [30], Alamri et al. (Saudi Arabia) (47.3%) [31], and Ghaleb et al. (Eastern Mediterranean Region) (52.7%) [18]. The majority of participants in our study were females (70.5%), in agreement with Elsaie et al. (Egypt) (76.6%) [29], while males more than females in Arafa et al. (Egypt and Saudi Arabia) (50.2%) [30] and Ghaleb et al. (Eastern Mediterranean Region) (51.2%) [18] studies. In Elkholy et al.’s (Egypt) [20] study, the male-to-female ratio was 1:1 (**S1 Table**).

Most of our participants, 87.1%, lived with their families; this is in line with the study of Ghaleb et al. (Eastern Mediterranean Region) [18], in which the proportion of participants who lived with their families was about 78.2%. According to our results, most participants were married (50.4%), in agreement with the literature (69.4% and 59.9%) as stated in Arafa et al. (Egypt and Saudi Arabia) [30] and Ghaleb et al. (Eastern Mediterranean Region) [18] studies, respectively. Most participants in our sample were pharmacists (33%) or physicians (22.1%). In comparison, most participants were physicians (48.4%, 47%, and 34.9%) in the studies by Arafa et al. (Egypt and Saudi Arabia) [30], Alamri et al. (Saudi Arabia) [31], and Ghaleb et al. (Eastern Mediterranean Region) [18] respectively.

According to our results, most participants (43.2%) had a work experience of two to five years, similar to the results of Elsaie et al. (Egypt) [29], where the "0–5 years" group represented 62.2% of participants and to Arafa et al. (Egypt and Saudi Arabia) [30] where the "1–5 years" of work experience group represented 44.4%. At the same time, Alamri et al. (Saudi Arabia) [31] showed that the "5–14 years" of work experience group represented the biggest group of the sample with 43.4%.

Based on our findings, only 18.4% of participants worked in the ICU, a similar percentage as in Arafa et al. (Egypt and Saudi Arabia) [30] with 19.7%. Our findings indicated that only 27.3% of participants had direct contact with COVID-19 cases. In contrast, Alamri et al. (Saudi Arabia) [31] study reported that about 47.6% of the participants had direct contact with COVID-19 cases. The other studies in the Arab region assessed anxiety and/or depression among healthcare workers during the pandemic and did not all report if the participants had direct contact with COVID-19 patients. The study by Elsaei et al. investigated dermatologists [29], and Arafa et al. included healthcare workers currently working in a hospital managing patients that were infected or might be infected with COVID-19 [30]. However, it remained unclear which proportion of healthcare workers in the Arafa et al. study had direct contact with COVID-19 patients [30]. Ghaleb et al. collected data from participants from two to three hospitals assigned to manage and treat patients with COVID-19 in each participating country [18]. However, if all the participants worked with patients, no further information was provided. On the other hand, Elkholy et al. reported that 40% of healthcare workers in direct contact with COVID-19 or suspected to have COVID-19 patients had moderate to severe anxiety [20]. At the same time, Alamri et al. reported that 75% of healthcare workers directly in contact with COVID-19 patients had anxiety, and 54% of healthcare workers in departments with no COVID-19 cases had anxiety [31].

### Interpretation of prevalence of anxiety

Regarding the prevalence of anxiety, 21.8% of pharmacists and 28.1% of physicians reported moderate to severe anxiety, respectively; this is lower than Arafa et al. (Egypt and Saudi Arabia) [30], in which 31.6% of physicians reported mild to moderate and 29.1% severe to very severe levels of anxiety, respectively. Previous literature [1, 32] discussed some causative factors that centered on sources of anxiety such as 1) availability of suitable personal protection equipment, 2) contracting COVID-19 at work and infecting their families at home, 3) lack of quick access to testing if they experience COVID-19 symptoms and concurrent worry of spreading the virus at work, 4) uncertainty on how their organization will support/take care of their requirements as individuals and as families should they contract the infection, 5) availability of day-care during extended working hours and school holidays, 6) assistance with additional personal and family requirements when workload and demand increase (food, hydration, lodging, transportation), 7) having the ability to offer quality medical treatment if stationed in a new region (e.g., non-ICU nurses having to function as ICU nurses), and 8) lack of access to current communication and information.

Based on the multivariable analysis, providing direct care to patients significantly predicted lower anxiety levels (Adjusted *P* = 0.008), in contrast to Alnazly et al. [33], which indicated that Jordanian medical staff who treated patients with positive COVID-19 infection experienced higher levels of anxiety than those who did not treat COVID-19-positive patients (*P* = 0.002). These findings corroborate those of a narrative review by Heath et al. [34], which demonstrated that assistance provided before and during an incident affected whether the medical personnel sustained injuries or experienced psychological distress. In the previous study [33], the anxiety of medical staffs was higher among those who treated COVID-19 patients. In our study, the health care workers providing direct care to the patients in general, not specifically for COVID-19 patients, were found to have a lower anxiety. The reason is unclear, however, lack of access to up-to-date information and communication was reported to be one of the contributing factors of anxiety among health care professionals [32]. There is a possibility that clinicians who are directly providing care to patients may have up to date COVID-19 information, management guidelines, and proper communication, which might in turn lesser anxiety among them. Further qualitative studies could be explained about the reasons for lowering anxiety among healthcare workers providing direct care to patients.

A positive attitude towards interprofessional teamwork and higher depressive symptoms significantly predicted higher anxiety levels (Adjusted *P* = 0.004 and <0.001, respectively). In comparison, Sakr et al. [35] study showed that high resilience significantly predicted higher anxiety levels (*P* = 0.011).

T.H. Alenazi et al. [36] study showed that age, being a male, being a cigarette smoker, having a chronic disease, being a nurse, being a medical laboratory technician, and having other specializations were significant predictors for severe anxiety (*P* = <0.0001, <0.0001, <0.0001, <0.0001, 0.02, 0.03, and 0.01, respectively). In contrast, our adjusted regression analysis showed that being a female, having different age groups (30–65), and being a nurse were no significant predictors for moderate and severe anxiety (Adjusted *P* = 0.065, 0.349, 0.964, and 0.980, respectively). While Sakr et al. [35] study showed an agreement with our findings as their results referred that being a female, age (> 35), and being a nurse were no significant predictors for moderate and severe anxiety (*P* = 0.368, 0.063, and 0.175, respectively).

Based on our results, being married was a significant predictor for moderate and severe anxiety (unadjusted *P* = 0.009), while T.H. Alenazi et al. [36] and Sakr et al. [35] studies showed that being married was not a significant predictor for anxiety levels (*P* = 0.88, 0.078, respectively).

### Interpretation of attitude toward interprofessional teamwork, risk perception, and coping strategies

Being married, living with friends, working as a tutor or medical representative, or working in the ICU significantly influenced a negative attitude toward interprofessional teamwork. On the other hand, more years of experience, providing direct care to patients, availability of mental health support at the workplace, and higher anxiety symptoms were significantly associated with a more positive attitude towards interprofessional teamwork. Surprisingly, positive attitude toward interprofessional teamwork showed a low correlation with anxiety symptoms (r = 0.38, *P <* 0.001). Generally, support and teamwork are strategies to reduce anxiety and depression among healthcare workers [37]. As a paradox, teamwork could enhance emotional attachment to team and increased demand for teamwork. This situation, sometimes, lead to higher commitment, engagement, and can lead to job-induced stress and anxiety [38]. Therefore, creating a nature of supportive teamwork while balancing the workload are providing degree of autonomy are crucial in the workplace of healthcare workers.

Our general linear model (GLM) of risk perception showed that being a female and having a mental health support team at the workplace were significant factors for risk perception toward COVID-19 (*P =* 0.038 and 0.001, respectively). At the same time, all items of current occupation and care for COVID-19 patients were non-significant factors for risk perception toward COVID-19 (*P >* 0.05). Our GLM analysis suggested that anxiety was the only significant factor affecting risk perception. Surprisingly, providing direct care to patients and working in the ICU did not significantly affect the risk perception of the study participants.

Based on our results, the most used coping strategies among our participants were positive thinking (66.8%), followed by family support (62.3%), and adequate sleep (56.8%). Htay et al. [26] showed that "getting family support" and "thinking positively" were listed as coping mechanisms by more than 70% of healthcare personnel when asked how they dealt with the COVID-19 epidemic. Nearly half of the respondents (58.4%) participated in worship and prayer, while 48.2% preferred getting enough rest and nourishment.

### Interpretation of prevalence of depression

Regarding the prevalence of depression, 34 pharmacists (25.6%) and 20 physicians (22.5%) had mild to severe depressive symptoms, compared to Arafa et al. (Egypt and Saudi Arabia) [30], which showed that about 35.4% and 38.4% of physicians had (mild to moderate) and (severe to very severe) depression, respectively. Being at the frontlines of healthcare, pharmacists have seen an increase in the number of patients, the number of screening and triage they perform, the COVID-19 information they deliver, the shortage of medications, and the workplace harassment they experience. These activities negatively impacted the pharmacists’ mental health and well-being, making them feel more stressed, burdened, and frustrated [39].

Before the COVID-19 pandemic, the prevalence of anxiety and depression reported in Egypt was 43.9% and 57.9% among about 164 medical students, respectively [40]. While among about 164 pharmacy students, the prevalence of anxiety and depression was 29.3% and 51.1%, respectively [40]. At the same time, about 66.7% of general practitioners reported high levels of burnout, while only 26.7% of specialists reported high burnout levels [41]. In Egypt in 2014, another study reported that participants who work in the internal medicine department scored the highest prevalence (69.64%), followed by surgeons (56.50%), hospital physicians (53.9%), family physicians (41.94%), and emergency physicians (39.39%) [42].

Multivariable logistic regression showed that having from two to five years of work experience, having highly or extremely problems and difficulties with daily life due to concerns about COVID-19, and having higher anxiety levels were significant predictors of depressive symptoms (*P* = 0.009, 0.010, and <0.001, respectively). Also, Yuan L et al. [43] findings of the logistic regression study showed that stress was linked to anxiety and depressive symptoms (*P* = 0.001 and 0.000, respectively). Depression and anxiety fall within the general category of internalizing illnesses [44]. According to estimates, patients with social anxiety disorder, patients with panic disorder, and patients with a generalized anxiety disorder had lifetime comorbidity with depression symptoms that ranged from (20% to 70%, 50%, and 43%, respectively) [45, 46].

Elgohary et al. [47] study showed that age and being female were significant predictors of depression (*P* = <0.001 and 0.03, respectively). In contrast, our results showed that that being a female and having different age groups (30–65) were no significant predictors for moderate and severe depression (Adjusted *P* = 0.118, 0439, and 0.739, respectively).

### Limitations and strength points

Our total responses were 403, which were similar to the total responses in most of the studies Elsaie et al. (Egypt) [29] (n = 415); Elkholy et al. (Egypt) [20] (n = 502); Arafa et al. (Egypt and Saudi Arabia) [30] (n = 275 from Egypt); Alamri et al. (Saudi Arabia) [31] (n = 389); and Ghaleb et al. (Eastern Mediterranean Region) [18] (n = 381 from Egypt) (**S1 Table**). The cross-sectional nature of the data limits the ability to draw valid conclusions about the association between the impact of the COVID-19 pandemic on healthcare workers and the resulting psychological consequences. The questionnaire was distributed in English, which might have caused a selection bias because potential participants with low English fluency were missed. Furthermore, the survey does not represent all the professions in the Egyptian healthcare system (generalisability is not applicable). Among the participants, there was a higher number of pharmacists and a relatively low number of nurses. Besides, the sample size calculation might only provide a rough estimate as no data from Egypt were available when we conducted the questionnaire. Therefore, the sample size was calculated using the prevalence of self-reported depressive symptoms among healthcare workers in China. In addition, English language proficiency was not checked.

## Conclusions

Our study identified helpful coping strategies among Egyptian healthcare workers during the COVID-19 pandemic. Surprisingly, providing direct care to patients and working in the ICU did not significantly affect the risk perception of our study participants. Higher depressive symptoms and a positive attitude towards interprofessional teamwork were significant predictors for higher anxiety levels in the study participants. Healthcare workers in contact with COVID-19 patients should receive adequate mental health support and follow-up if necessary.

We recommend that healthcare workers seek wide-scale mental health screening and that decision-makers implement public health campaigns. Furthermore, healthcare authorities should support population-level efforts to improve the prevention and treatment strategies for mental health problems.

To conclude, the availability of mental health facilities at the workplace could improve interprofessional teamwork and reduce the risk perception towards emerging public health-related events.

## Supporting information

S1 Table Summary of similar literature (*N = 7*). (DOCX)

S1 Data (XLSX)

## List of abbreviations

AMU: Asia Metropolitan University
ARDS: Acute Respiratory Distress Syndrome
COVID-19: Coronavirus SARS-CoV-2
GAD-7 scale: Generalized Anxiety Disorder (GAD-7) scale
GLM: General linear regression model
MERS-CoV: The Middle East respiratory syndrome coronavirus
PHQ-9: Patient Health Questionnaire
SARS: Severe Acute Respiratory Syndrome
GLM: General Linear Model
