# Supplementary material for: Mental health, risk perception, and coping strategies among healthcare workers in Egypt during the COVID-19 pandemic
Source: PLoS One. 2023 Feb 27;18(2):e0282264. doi: 10.1371/journal.pone.0282264 (PMC9970061; doi:10.1371/journal.pone.0282264)
Supplement: S1 Table — (DOCX) [file pone.0282264.s001.docx]

**Supplementary Table 1:** Summary of Similar Literature (*N = 7*)

| Study ID | Country | Total Responses | Most of the Sample Age (Years) | Gender | Mental Health Assessment | Scale | Target Population |
| --- | --- | --- | --- | --- | --- | --- | --- |
| Elsayed et al. (Our Study) | Egypt | 403 | 26 – 40 (70.7%) | Male = 119 (29.5%) Female = 284 (70.5%) | Anxiety  Depression  Risk Perception Questions  Coping | GAD-7, PHQ-9, Risk Perception, Coping, and Teamwork Questions | Healthcare Workers |
| Elsaie et al. | Egypt | 415 | 30 – 40 (65.5%) | Females = 318 (76.6%)  Males 97 (23.4%) | Depression  Anxiety  Stress | DASS-21 | Egyptian Dermatologists |
| Elkholy et al. | Egypt | 502 | 31 – 40 (44.4%) | The male-to-female ratio was 1:1 | Depression  Anxiety  Insomnia  Stress | GAD-7, PHQ-9, ISI, and PSS | Frontlines Healthcare Workers |
| Arafa et al. | Egypt and Saudi Arabia | 426 (275 from Egypt and 151 from Saudi Arabia) | 18 – 30 (47.2%) | Male = 214 (50.2%) Female = 212 (49.8%) | Depression  Anxiety  Stress | DASS-21 | Frontlines Healthcare Workers |
| Alamri et al. | Saudi Arabia | 389 | 30 – 39 (47.3%) | Male = 266 (68.4%) Female = 123 (31.6%) | Depression  Anxiety | GAD-7 and PHQ-9 | Healthcare Workers |
| Kamberi et al. | Albania | 410 | 26 – 40 (46.3%) | Male = 67 (16.3%) Female = 322 (78.5%) | Anxiety  Depression  Risk Perception Questions  Coping | GAD-7, PHQ-9, Risk Perception, and Coping Questions | Healthcare Workers |
| Rillera Marzo et al. | Philippines | 516 | 41 – 60 (Doctors: 229 (54.14%) and Nurses: 38 (43.68%)) | Male = 230 (44.58%) Female = 286 (55.42%) | Anxiety  Depression  Risk Perception Questions  Coping | GAD-7, PHQ-9, Risk Perception, and Coping Questions | Healthcare Workers |
| Ghaleb et al. | Eastern Mediterranean Region | 1448 from Iraq (n = 381), Egypt (n = 308), Somalia (n = 212), Sudan (n = 202), Yemen (n = 160), Jordan (n = 55), Pakistan (n = 53), Afghanistan (n = 36), and Morocco (n = 41). | ≤ 30 (52.7%) | Male = 742 (51.2%) Female = 706 (48.8%) | Depression  Anxiety  Stress  Psychological distress | DASS-21 and CPDI | Healthcare Workers |

Abbreviations: Patient Health Questionnaire (PHQ-9); Generalized Anxiety Disorder (GAD-7); Insomnia Severity Index (ISI); Perceived Stress Scale (PSS); Hospital Anxiety and Depression Scale (HADS); Health Anxiety Inventory (HAI); Depression, Anxiety, and Stress Scale (DASS-21); and COVID-19 Peritraumatic Distress Index (CPDI)
